# Diagnostic role of hepatobiliary scintigraphy in bile leak evaluation: A systematic review and meta-analysis

**DOI:** 10.22038/aojnmb.2025.89502.1650

**Published:** 2026

**Authors:** Jasim Jaleel1, Mangu Srinivas Bharadwaj, Bangkim Chandra Khangembam, Suhana Sulfiker, Ananthu S J Narayan

**Affiliations:** 1 Department of Nuclear Medicine, Institute of Liver and Biliary Sciences, New Delhi, India; 2 Department of Nuclear Medicine, All India Institute of Medical Sciences, New Delhi, India; 3 Department of Palliative Medicine, All India Institute of Medical Sciences, New Delhi, India; 4 Department of Hepatology, Institute of Liver and Biliary Sciences, New Delhi, India

**Keywords:** Hepatobiliary scintigraphy, Bile leak, Diagnostic accuracy Abdominal trauma, Cholecystectomy A B S T R A C T

## Abstract

**Objective(s):**

This study aimed to systematically evaluate the diagnostic accuracy of hepatobiliary scintigraphy (HBS) for detecting bile leaks in post-traumatic and postoperative settings, given the increasing incidence of such complications following hepatobiliary surgeries and abdominal trauma.

**Methods:**

A systematic review and meta-analysis were conducted in accordance with the PRISMA guidelines. Literature searches of PubMed, Embase, and Scopus databases were performed through June 19, 2025. Studies were included if they assessed patients with suspected bile leak using HBS and reported sufficient data to construct diagnostic contingency tables. The quality of included studies was assessed using the QUADAS-2 tool. Pooled sensitivity, specificity, positive predictive value (PPV), negative predictive value (NPV), and overall diagnostic accuracy were calculated. Subgroup analyses evaluated diagnostic performance across clinical contexts such as post-trauma, liver transplant/resection, and cholecystectomy.

**Results:**

Sixteen studies with 673 patients were included. The pooled sensitivity and specificity of HBS were 0.882 (95% CI: 0.81–0.93) and 0.93 (95% CI: 0.83–0.97), respectively. The pooled PPV was 0.874 and NPV was 0.965, with an area under the SROC curve of 0.94, indicating excellent diagnostic performance. Subgroup analyses showed the highest accuracy in trauma patients, while specificity varied more in postoperative settings, particularly after liver transplant or resection.

**Conclusion:**

HBS is a highly sensitive and reliable imaging modality for ruling out bile leaks. While variability in specificity warrants cautious interpretation in complex surgical cases, HBS should be considered a valuable first-line, non-invasive diagnostic tool in evaluating suspected bile leaks.

## Introduction

 In recent years, the number of hepatobiliary surgeries, particularly laparoscopic cholecyst-ectomies and adult living donor liver transplantations, has steadily increased. (1). This rise has been accompanied by a corresponding increase in postoperative complications, most notably bile leaks (2). 

 While iatrogenic bile duct injuries are well-documented, bile leaks resulting from blunt abdominal trauma present a more complex and often subtle clinical challenge(3). Prompt and accurate diagnosis of bile leaks is critical, as delays can result in patient morbidity and mortality (4). In this context, diagnostic imaging plays a pivotal role in the early detection and effective management of bile leaks (5).

 Computed tomography (CT) is often the first-line imaging modality in trauma settings, but its findings in post-traumatic biliary injury are frequently subtle and nonspecific (6). While CT can identify liver lacerations, peri- or intrahepatic fluid collections, and ascites, these signs are indirect and require a high index of suspicion for bile duct injury (7). Progressive increase in size of a well-circumscribed, low-attenuation perihepatic or intraparenchymal collection may suggest a biloma, while persistent or increasing low-attenuation intraperitoneal fluid post-trauma raises concern for bile leakage (8). Ultrasonography (USG) is typically used for follow-up and can identify complications such as bilomas, gallbladder wall thickening, and ascites (9).

 Endoscopic Retrograde Cholangiopancreato-graphy (ERCP) offers both diagnostic and therapeutic capabilities. It can localize the site of biliary leak and facilitate management through sphincterotomy and stent placement, reducing pressure within the biliary system to promote healing (10). However, ERCP is invasive and carries risks such as pancreatitis and bleeding, making it less suitable as a first-line diagnostic tool (11). Magnetic Resonance Cholangiopancreatography (MRCP) with hepatobiliary contrast agents offers a noninvasive method for assessing both functional and anatomical aspects, allowing for the detection of active bile leaks through contrast extravasation (12). Despite its advantages, MRCP may be limited by poor bile duct filling in reduced hepatic function, contraindications in patients with certain implants or foreign bodies, and artifacts from motion or surgical materials (13).

 Hepatobiliary Scintigraphy (HBS), also known as cholescintigraphy, is a non‑invasive nuclear medicine imaging technique in which a ^99m^Technetium (^99m^Tc)-labeled iminodiacetic acid (IDA) radiotracer, such as lidofenin [HIDA], iprofenin [PIPIDA], disofenin [DISIDA], and mebrofenin, is injected intravenously and rapidly taken up by hepatocytes before being excreted into bile (14). This dynamic tracing provides real‐time functional visualization of bile flow through the liver, gallbladder, and bile ducts, thus offering physiologic insight that purely anatomical modalities cannot (15). It is particularly valuable for early detection and localization of bile leaks, since tracer extravasation outside the biliary tree signals leakage, and it can even quantify leak volume (16). Among the tracers, Tc‑99m mebrofenin is the current clinical favorite due to its superior hepatic extraction fraction (~98 % vs ~88 % for others), faster hepatic clearance, minimal renal excretion, and interpretable even in hyperbilirubinemia (17). These tracers allow HBS to non-invasively assess hepatocyte function, gallbladder ejection fraction, biliary 

obstruction, leaks, and deliver functional data critical for diagnosis, postoperative monitoring, and surgical planning (15).

 In HBS, the detection of a bile leak is based on the visualization of radiotracer accumulation outside the confines of the biliary system, indicating extravasation of bile into the peritoneal cavity (18). Radiotracer is extracted by functioning hepatocytes and excreted into the bile ducts, which sequentially progress from the liver to the intrahepatic ducts, through the common bile duct, into the gallbladder (if the cystic duct is patent), and finally into the small bowel (19). In the setting of a bile leak, the radiotracer can be seen leaking into abnormal areas, most commonly the subhepatic space, Morrison's pouch, or peritoneal cavity. On dynamic imaging, early signs of a bile leak may include delayed tracer entry into the bowel and persistent focal accumulation of activity outside the expected biliary tract. Delayed static images, acquired up to several hours post-injection, may enhance the visualization of subtle leaks (20). 

 SPECT/CT significantly improves localization by correlating functional findings with anatomical detail, helping differentiate true leaks from adjacent bowel activity or fluid collections, and also correctly pinpointing the site of bile leak (21–23). The sensitivity of hepatobiliary scintigraphy for bile leaks is high, particularly for low-output leaks that may be missed on cross-sectional imaging (24). Findings may also be used to assess the severity of the leak and monitor response to conservative or surgical management (25).

 Although numerous individual studies, case reports, and case series exist on HBS for detecting bile leaks, the evidence remains fragmented and methodologically inconsistent. These studies often vary significantly in terms of patient populations, imaging protocols, radiotracer selection, and clinical indications, making it challenging to draw generalizable conclusions. Furthermore, the absence of a comprehensive synthesis of diagnostic accuracy metrics limits the clinical applicability of existing findings. Inconsistencies across literature contribute to uncertainty in clinical decision-making and hinder the development of standardized imaging guidelines. As such, a systematic review and meta-analysis is essential to consolidate the available evidence, quantify the diagnostic performance of HBS, and provide clinicians with reliable, evidence-based recommendations for evaluating suspected bile leaks.

## Methods

### Systematic search strategy

 A systematic literature search was conducted in PubMed, Scopus, and Embase from inception to June 19, 2025. All languages were considered without restriction. The search strategy was tailored to each database using appropriate keywords and MeSH terms relevant to the research question. A PRISMA flow diagram outlines the screening and selection process in detail.

### Ethical approval

 This study is a systematic review and meta-analysis of previously published data and does not involve any new studies with human participants or animals performed by the authors. Hence, ethical approval and informed consent were not required.

### Inclusion criteria

 Studies were eligible for inclusion if they involved patients with suspected bile leak following trauma or surgery, and underwent hepatobiliary scintigraphy to assess diagnosis. 

 The included studies were required to provide sufficient data to construct a contingency table (true positives, false positives, false negatives, and true negatives). A definitive diagnosis had to be established by endoscopic, radiological, or clinical evaluation.

### Data extraction

 Data extraction was performed independently by two reviewers (J.J. and M.S.B.), with any discrepancies resolved by mutual consensus. Data were systematically extracted from each included study, capturing the first author, year of publication, type of radiotracer used, patient cohort size, and reported diagnostic outcomes. 

 Extracted data allowed for the calculation of pooled sensitivity, specificity, Positive Predictive Value (PPV), Negative Predictive Value (NPV), and overall diagnostic accuracy.

### Quality assessment of included studies

 The Quality Assessment of Diagnostic Accuracy Studies 2 (QUADAS-2) tool was used to assess the methodological quality of all included studies. Any disagreements between the two reviewers were resolved by consensus. 

 Risk of bias and concerns regarding applicability were evaluated across the domains of patient selection, index test, and reference standard. At the same time, flow and timing were assessed exclusively for risk of bias.

### Statistical analysis

 To evaluate the diagnostic performance across studies, pooled estimates of sensitivity and specificity were calculated. Summary Receiver Operating Characteristic (SROC) curves were constructed, and the Area Under the Curve (AUC) was determined to summarize overall diagnostic accuracy. Heterogeneity among studies was quantified using Higgins’ I² statistic, and publication bias was calculated using Deeks’ funnel plot asymmetry test. To explore potential sources of heterogeneity, subgroup analyses were conducted by categorizing the included studies into three distinct groups. 

 Statistical analyses were performed using “meta”, “meta4diag”, and “metafor” packages in R software (version 4.5.1; R Foundation for Statistical Computing).

## Results

**Figure 1:**
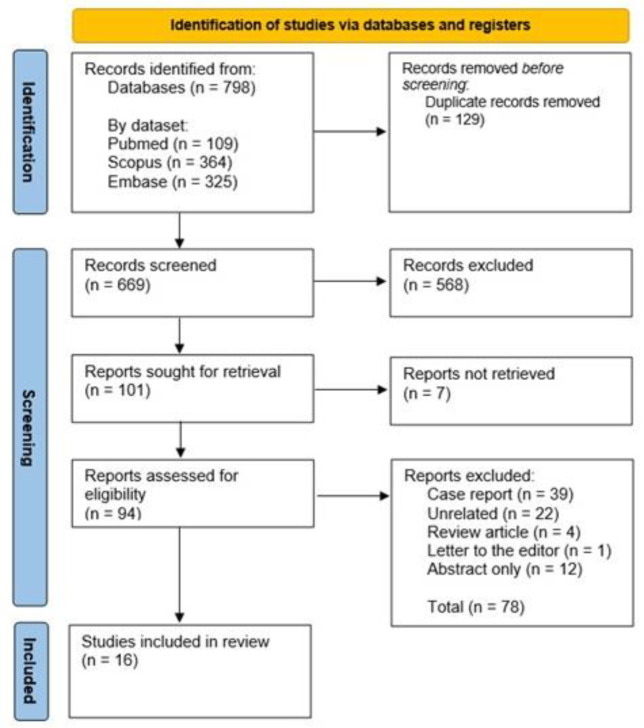
PRISMA Flow Diagram showing the results of the database search for publications assessing the diagnostic accuracy of HBS in bile leak

 The study selection process adhered to the Preferred Reporting Items for Systematic Reviews and Meta-Analyses (PRISMA) guidelines (Figure 1). An initial systematic search identified 798 articles. Following the removal of 129 duplicates, 669 unique records underwent title and abstract screening, resulting in the exclusion of 568 articles that did not meet the inclusion criteria. Of the 94 full-text articles assessed for eligibility, 78 were excluded due to being case reports, unrelated topics, review articles, letters to the editor, or abstracts only. Ultimately, 16 studies were included in the final meta-analysis.

 The characteristics of the included studies are listed in Table 1. A total of 16 studies with 673 patients were included in our analysis. Three studies included post-traumatic patients, while twelve included post-surgery patients, and one study included both post-surgery and post-traumatic patients. Surgeries included open and laparoscopic cholecystectomy, liver resection, and liver transplantation. 

**Table 1:** Baseline characteristics of included studies

**Authors**	**Country**	**Year**	**Nature of study**	**Tracer**	**Mean dose**	**SPECT/CT done**	**Total patients**	**Indication**	**Type of surgery**
Sty el al (26)	USA	1982	Prospective	PIPIDA or Disofenin	4 mCi	No	8	Post trauma	
Creutzig et al (37)	Germany	1984	Prospective	IDA	Not Reported	No	134	Post-surgery	Post liver resection/liver transplantation
Ryttov et al (27)	Denmark	1989	Prospective	IDA	Not Reported	No	10	Post-surgery	Cholecystectomy
Mochizuki et al (38)	USA	1991	Prospective	Mebrofenin	5 mCi	No	55	Post-surgery	liver transplantation
Walker et al (39)	USA	1992	Retrospective	DISIDA	4 mCi	No	5	Post-surgery	Cholecystectomy
Pasmans et al (28)	Netherlands	1991	Prospective	IDA	3.5 mCi	No	51	Post-surgery	Cholecystectomy
Brooks et al (40)	USA	1993	Prospective	DISIDA	Not Reported	No	8	Post-surgery	Cholecystectomy
Banzo et al (32)	Spain	1997	Prospective	Mebrofenin	Not Reported	No	20	Post-surgery	liver transplantation
Hasl et al (41)	USA	2000	Prospective	Mebrofenin	Not Reported	No	71	Post-surgery	cholecystectomy
Kim et al (42)	South Korea	2002	Prospective	DISIDA	5 mCi	No	79	Post-surgery	liver transplantation
Fleming et al (29)	USA	2005	Retrospective	Mebrofenin	4 mCi	No	39	Post trauma	
Mittal et al (30)	India	2008	Retrospective	Mebrofenin	Not Reported	No	35	Post trauma	
Sofayan et al (33)	Saudi Arabia	2008	Prospective	Disofenin	5 mCi	No	34	Post-surgery	liver transplantation
Sharma et al (22)	India	2012	Retrospective	Mebrofenin	Not Reported	Yes	32	Post-surgery in 26, Post trauma in 6	
Eckenschwiller et al (34)	Germany	2016	Retrospective	Mebrofenin	Not Reported	No	42	Post-surgery	Post liver resection/liver transplantation
Bickel et al (31)	Israel	2023	Prospective	IDA	5 mCi	No	50	Post-surgery	Cholecystectomy

**Figure 2:**
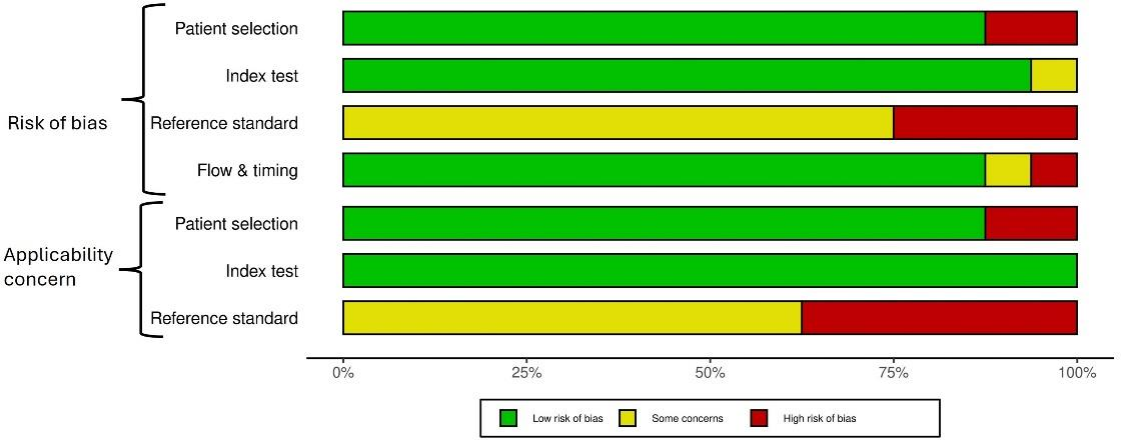
Summary of risk of bias and applicability concerns using the QUADAS 2 score

**Figure 3:**
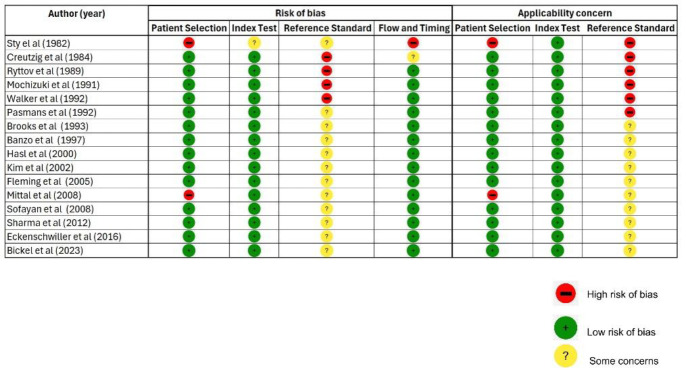
Detailed risk of bias and applicability concerns using the QUADAS 2 score for individual studies

 The overall methodological quality of the 16 included studies was assessed using the QUADAS-2 tool (Figure 2), with results ranging from moderate to good. Fourteen of the sixteen studies satisfied at least 3 of the 4 QUADAS-2 domains, reflecting generally acceptable internal validity (Figure 3). For the patient selection domain, most studies were prospective and enrolled consecutive patients, representative of routine clinical populations; however, five studies presented a high risk due to their retrospective design and selective inclusion criteria, which could introduce bias and limit applicability. In the index test domain, the majority demonstrated a low risk of bias, with standardized imaging protocols and blinded interpretation commonly reported; however, a few lacked explicit blinding procedures. The reference standard domain exhibited the most significant methodological limitation, as all studies relied on heterogeneous diagnostic confirmation (e.g., surgical findings, clinical follow-up, imaging), and none consistently applied blinding, resulting in a high risk of bias and significant applicability concerns across the board. In the flow and timing domain, most studies had low risk due to complete follow-up and consistent diagnostic evaluation; however, some did not report the timing between index and reference tests. Despite variability in the rigor of the reference standards, the included studies provide valuable real-world insights into the diagnostic performance of hepatobiliary scintigraphy for detecting bile leaks.

**Figure 4:**
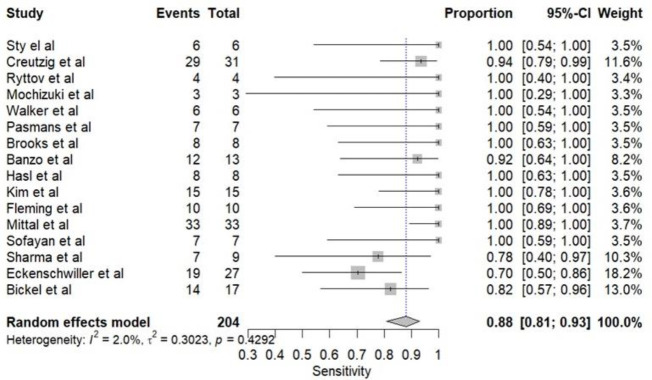
Forest plot showing pooled sensitivity of HBS in detecting bile leak

**Figure 5:**
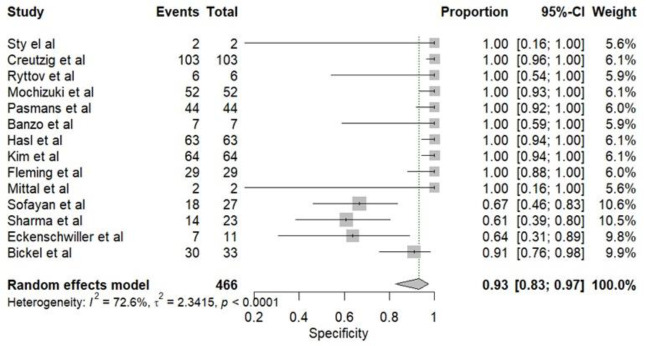
Forest plot showing pooled specificity of HBS in detecting bile leak

**Figure 6:**
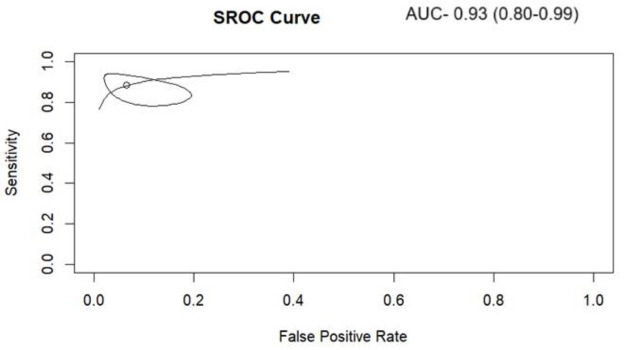
Hierarchical summary ROC curves for the detection of bile leak on HBS

 The pooled diagnostic performance of HBS for detecting bile leaks across the included studies was high. The pooled sensitivity (Figure 4) was 0.882 (95% CI: 0.81–0.93), and the pooled specificity (Figure 5) was 0.93 (95% CI: 0.83-0.97), suggesting strong discriminatory ability. The pooled Positive Predictive Value (PPV) was 0.8744 (95% CI: 0.73–0.98), while the pooled Negative Predictive Value (NPV) was notably higher at 0.96 (95% CI: 0.90–0.99), indicating a robust capacity to rule out bile leaks. The overall pooled diagnostic accuracy was 0.94 (95% CI: 0.86–0.98), and the Area Under the Curve (AUC) of the Summary Receiver Operating Characteristic curve (SROC) was 0.94 (95% CI: 0.80-0.99), supporting excellent test performance (Figure 6). 

**Figure 7:**
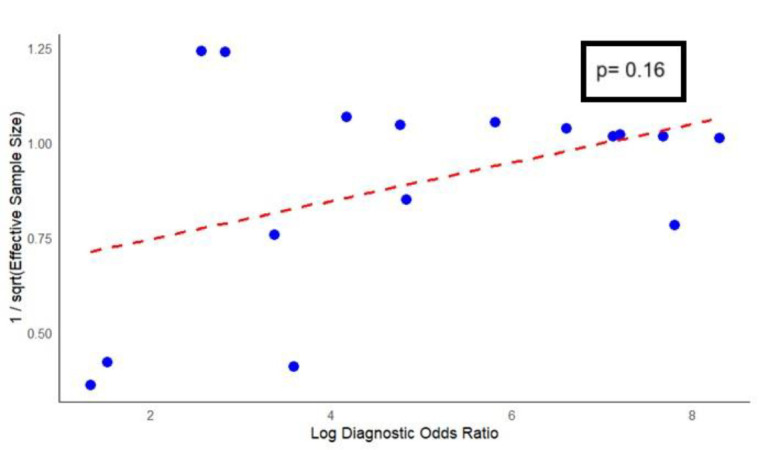
Results of Deeks’s funnel plot of the asymmetry test for publication bias

 Assessment of heterogeneity using Higgins’ I² statistic revealed no observed heterogeneity for sensitivity (I²=0; 95% CI: 0-0.94) and substantial heterogeneity for specificity (I²=0.78; 95% CI: 0.18-0.99). Evaluation of publication bias using Deeks’ funnel plot asymmetry test (Figure 7) indicated no statistically significant evidence of bias among the included studies (p=0.16).

 We performed subgroup meta-analyses (Table 2) to evaluate sensitivity and specificity across three clinical groups: post-abdominal trauma (Group 1), post-liver transplantation or liver resection (Group 2), and post-cholecystectomy (Group 3). One study (Sharma et al(22)) was not included as it contained both post-traumatic and post-surgery cases. Excellent and consistent sensitivity and specificity in the post-abdominal trauma group were seen. Sensitivity remains high but slightly more variable in post-liver transplant/ resection and post-cholecystectomy groups. At the same time, specificity shows greater variability in these latter groups, particularly after liver transplant or resection, as indicated by high heterogeneity statistics. 

**Table 2:** Subgroup analysis of the three different groups

**Group**	**No of studies**	**Sensitivity** **(95% CI)**	**i2 heterogeneity** ** (** ***P*** ** value)**	**Specificity** **(95% CI)**	**i2 heterogeneity ** **(** ***P*** ** value)**
Group 1 (post-abdominal trauma)	3	0.98 (0.94-1.0)	0 (0.78)	0.98 (0.93-1.0)	0 (0.62)
Group 2 (Post-liver transplant/ liver resection)	6	0.90 (0.82-0.98)	0.57 (0.09)	0.87 (0.72-1.0)	0.98 (0.01)
Group 3- post-cholecystectomy	6	0.89 (0.81-0.97)	0 (0.75)	0.93 (0.84-1.0)	0.96 (0.01)

 These findings highlight the generally robust diagnostic performance of the test across clinical contexts but suggest caution in interpreting specificity in liver transplant/ resection and cholecystectomy populations due to substantial inter-study variability.

 SPECT/CT was performed only in one study by Sharma et al (22). In their study, the diagnostic performance of SPECT-CT was markedly superior, demonstrating a sensitivity of 88.8%, specificity of 100%, and an overall accuracy of 96.8%. In contrast, planar HBS yielded lower values, with a sensitivity of 77.7%, specificity of 60.8%, and accuracy of only 65.6%. The difference in diagnostic accuracy between the two modalities was statistically significant (65.6% vs. 96.8%; *P*=0.021). Planar HBS produced nine false-positive results, whereas SPECT-CT not only avoided such errors but also correctly identified the precise site of bile leakage in eight of nine cases. Receiver operating characteristic (ROC) analysis further confirmed that observer confidence was significantly higher with SPECT-CT compared to planar imaging (P=0.045).

 Several included studies provided information on the severity of bile leaks and associated outcomes. Early series such as Sty et al. (26) demonstrated that contained subhepatic leaks often resolved spontaneously, whereas large intraperitoneal leaks led to bile peritonitis and required surgical repair. Ryttov et al. (27) similarly showed that patients with minimal subhepatic accumulation and preserved bowel activity had an uneventful course, while those with complete extravasation and absent bowel visualization uniformly required reoperation. Pasmans et al. (28) reported that patients with minor scintigraphic tracer accumulation in the paracolic gutter recovered without sequelae, while those with more extensive leaks developed symptoms and underwent surgical revision. Fleming et al. (29) confirmed that patients with contained leaks or bilomas were managed conservatively, whereas free intra-peritoneal leaks necessitated multiple interventions and longer hospitalization.

 More recent studies highlighted the impact of scintigraphy on management decisions. Mittal et al. (30) demonstrated that localized leaks following trauma were successfully managed conservatively, while active or free leaks mandated surgical repair. Bickel et al. (31) prospectively showed that patients with fair duodenal transit and minimal/no leak recovered uneventfully, whereas those with significant leaks or impaired bowel transit required ERCP or surgery. Banzo et al. (32) also noted that positive HBS findings led to targeted interventions, while negative scans prevented unnecessary invasive procedures. In the transplant setting, Sofayan et al. (33) and Eckenschwiller et al. (34) confirmed that scintigraphy findings guided ERCP, drainage, or surgical revision, with accuracy varying between resection and transplant groups. 

 Sharma et al. (22) further demonstrated that SPECT-CT accurately localized the leak site, allowing precise surgical or endoscopic planning. Collectively, these studies indicate that beyond diagnosis, the extent and distribution of tracer leakage, as well as duodenal transit, stratify prognosis and guide tailored management strategies ranging from conservative follow-up to endoscopic stenting or operative repair.

## Discussion

 This meta-analysis demonstrates that HBS exhibits high diagnostic accuracy in detecting bile leaks across various clinical settings. The pooled sensitivity and specificity were 0.882 (95% CI: 0.81–0.93) and 0.93 (95% CI: 0.83–0.97), respectively, reflecting strong discriminatory ability. The overall diagnostic accuracy was high (0.94; 95% CI: 0.86–0.98), and the AUC under SROC was 0.94 (95% CI: 0.80–0.99), indicating excellent diagnostic efficacy. Notably, the pooled NPV was 0.97 (95% CI, 0.90–0.99), underscoring the reliability of HBS in ruling out bile leaks. Subgroup analyses further confirmed the robustness of HBS performance across varying clinical contexts, including post-abdominal trauma, liver resection or transplantation, and post-cholecystectomy, with exceptionally consistent sensitivity observed in trauma-related cases. While specificity showed greater variability in surgical subgroups, the overall findings support the dependable utility of HBS in both traumatic and postoperative settings.

 The high NPV suggests that a negative HBS result can confidently exclude a bile leak, thereby reducing the need for further invasive diagnostics in many cases. This has significant implications for patient management, particularly following hepatobiliary surgery or abdominal trauma, where early and accurate diagnosis is essential to minimize morbidity (4). 

 The strong diagnostic performance of HBS also supports its use as a non-invasive first-line imaging modality, potentially decreasing reliance on more invasive procedures, such as ERCP, or costly imaging, like contrast-enhanced MRI. Incorporating HBS into routine diagnostic algorithms may therefore enhance diagnostic efficiency, reduce procedural risks, and streamline patient care in settings where bile leaks are suspected.

 Subgroup analysis revealed essential differences in the diagnostic performance of HBS across clinical contexts. In patients with post-abdominal trauma, HBS demonstrated consistently high sensitivity and specificity, supporting its reliability in this setting. This strong performance may reflect the relatively clear clinical context and more defined timing of bile leak onset in traumatic cases, which facilitates accurate imaging and interpretation. 

 In contrast, while HBS maintained high sensitivity in the post-liver transplantation or liver resection subgroup, specificity was more variable. A similar pattern was observed in the post-cholecystectomy group, where sensitivity remained good, but specificity was less consistent across studies. This variability in specificity may be attributed to several factors. 

 The differences in imaging protocols, such as tracer type, administered dose, and timing of scan acquisition, could influence detection accuracy. The heterogeneity in patient populations, including variations in baseline liver function (34), surgical technique (22), and severity of underlying pathology, may also impact imaging interpretation (16). Finally, inconsistencies in the timing of HBS relative to the surgical procedure could contribute to diagnostic variability; early or delayed imaging may miss evolving leaks or confound findings with postoperative changes. These factors highlight the need for standardized imaging protocols and patient stratification to enhance diagnostic precision in postoperative bile leak evaluation.

 The methodological quality of the included studies was generally acceptable, with several notable strengths. The majority were prospective in design, enhancing the reliability of patient selection and outcome assessment. Most studies also reported blinded interpretation of hepatobiliary scintigraphy results, reducing the risk of interpretation bias. Furthermore, standardized imaging protocols were commonly employed, supporting the internal validity of diagnostic assessments.

 We also observed that most studies except one (22) did not utilize SPECT/CT for precise localization, which may impact the specificity of the findings. SPECT/CT enhances the diagnostic accuracy of hepatobiliary scintigraphy by providing precise anatomical localization of functional abnormalities. It aids not only in the identification of false-positive results (22) but also in the characterization of biliary collections, including assessment of the severity and extent of bile leaks and localization of the exact site of leakage for therapeutic planning, such as drain placement or surgical intervention (21). Moreover, SPECT/CT helps to differentiate intra-abdominal fluid collections (e.g., bilomas vs abscesses or seromas), clarifies the relationship of tracer activity to surgical anatomy or drains, and improves interpretation in postoperative settings where altered anatomy may confound planar imaging (35). Its ability to correlate tracer distribution with cross-sectional imaging improves confidence in diagnosing conditions such as biliary obstruction, bile peritonitis, and retained biliary stents or clips, ultimately facilitating more targeted and timely clinical management (36).

 A subset of studies employed retrospective designs, which introduce potential selection bias and limit the generalizability of the findings. Additionally, reference standards varied considerably across studies; some relied solely on clinical follow-up, while others used imaging findings or surgical confirmation. This inconsistency may have influenced the accuracy of diagnostic classification. Moreover, none of the studies applied blinding to reference standard assessments, raising concerns about detection bias. Finally, although sensitivity was consistently high, substantial heterogeneity was observed in specificity estimates, particularly in postoperative subgroups. This heterogeneity may have influenced the pooled specificity, highlighting the need for more uniform methodological approaches in future diagnostic accuracy studies.

 This study poses a few limitations. While this meta-analysis offers valuable insights into the diagnostic performance of hepatobiliary scintigraphy (HBS) for detecting bile leaks, several limitations should be considered. First, the number of studies within individual clinical subgroups was relatively small, which may limit the statistical robustness of subgroup comparisons. Second, there was notable variability in study quality and methodologies, including differences in imaging protocols, reference standards, and study design, which could affect the consistency and generalizability of pooled estimates. Third, potential underreporting of negative or inconclusive results may have skewed the evidence base, despite Deeks’ funnel plot asymmetry test showing no statistically significant publication bias (p=0.16). However, the test’s power to detect bias is limited in meta-analyses with a small number of studies. These factors suggest that while the overall findings are encouraging, they should be interpreted with caution, and further high-quality, standardized research is warranted. Another limitation is the inability to separately analyze post-liver transplant and post-liver resection cohorts, as few included studies (37) did not provide disaggregated data.

 Future research should focus on developing and validating standardized diagnostic pathways for evaluating suspected bile leaks, particularly in postoperative and trauma settings. Prospective, multicenter studies using uniform imaging protocols and reference standards are essential to improve methodological consistency and enhance the reliability of diagnostic accuracy estimates. 

 Additionally, research should aim to include more diverse clinical populations across various healthcare settings to reflect real-world variability better and ensure the broader applicability of findings. These efforts will be crucial to optimizing the role of hepatobiliary scintigraphy in clinical decision-making algorithms and enhancing patient outcomes.

## Conclusion

 Hepatobiliary scintigraphy demonstrates high diagnostic accuracy for detecting bile leaks, with particularly strong sensitivity and negative predictive value, making it a reliable modality for ruling out bile leaks in both posttraumatic and postoperative settings. However, while sensitivity remains consistently high, variability in specificity, especially in complex surgical populations such as those undergoing liver transplant or resection, warrants cautious interpretation. Clinicians should consider these limitations when integrating HBS into diagnostic pathways and, where appropriate, complement it with additional imaging or clinical correlation in high-risk cases.
